# Patch Attention Layer of Embedding Handcrafted Features in CNN for Facial Expression Recognition

**DOI:** 10.3390/s21030833

**Published:** 2021-01-27

**Authors:** Xingcan Liang, Linsen Xu, Jinfu Liu, Zhipeng Liu, Gaoxin Cheng, Jiajun Xu, Lei Liu

**Affiliations:** 1 Hefei Institutes of Physical Science, Chinese Academy of Sciences, Hefei 230031, China; lxcan@mail.ustc.edu.cn (X.L.); liujinfu@mail.ustc.edu.cn (J.L.); liuzhipeng@mail.ustc.edu.cn (Z.L.); ba181681@mail.ustc.edu.cn (G.C.); 2 University of Science and Technology of China, Hefei 230026, China; jiajun@mail.ustc.edu.cn (J.X.); liulei95@mail.ustc.edu.cn (L.L.); 3 Anhui Province Key Laboratory of Biomimetic Sensing and Advanced Robot Technology, Hefei 230031, China

**Keywords:** facial expression recognition, patch attention, shallow feature, feature extraction, facial representation, convolutional layer

## Abstract

Recognizing facial expression has attracted much more attention due to its broad range of applications in human–computer interaction systems. Although facial representation is crucial to final recognition accuracy, traditional handcrafted representations only reflect shallow characteristics and it is uncertain whether the convolutional layer can extract better ones. In addition, the policy that weights are shared across a whole image is improper for structured face images. To overcome such limitations, a novel method based on patches of interest, the Patch Attention Layer (PAL) of embedding handcrafted features, is proposed to learn the local shallow facial features of each patch on face images. Firstly, a handcrafted feature, Gabor surface feature (GSF), is extracted by convolving the input face image with a set of predefined Gabor filters. Secondly, the generated feature is segmented as nonoverlapped patches that can capture local shallow features by the strategy of using different local patches with different filters. Then, the weighted shallow features are fed into the remaining convolutional layers to capture high-level features. Our method can be carried out directly on a static image without facial landmark information, and the preprocessing step is very simple. Experiments on four databases show that our method achieved very competitive performance (Extended Cohn–Kanade database (CK+): 98.93%; Oulu-CASIA: 97.57%; Japanese Female Facial Expressions database (JAFFE): 93.38%; and RAF-DB: 86.8%) compared to other state-of-the-art methods.

## 1. Introduction

In our daily life, we communicate with each other not only in words, but also in many other nonverbal ways such as body language, intonation, and facial expressions. As the famous psychologist Mehrabian said, facial expressions convey 55% of a communicated message, which is more than the part conveyed by the combination of voices and languages [1]. Therefore, understanding the unspoken words from a person’s facial expression is a fundamental human trait. As people, we can presume the state of someone’s emotion by observing their face, but if we were machines, we only could utilize an automatic algorithm for emotion recognition. For this reason, automatic facial expression recognition (FER), which has attracted much more attention in recent years, is an interesting and challenging problem, and has become prevalent in a broad range of applications such as driver fatigue surveillance [2], smile or pain detection [3,4], social media [5], interpersonal relation prediction [6], and human–computer interaction [7,8].

Facial expressions can be divided into six basic emotions, namely, anger (An); disgust (Di); fear (Fe); happiness (Ha); sadness (Sa); surprise (Su); and one neutral (Ne) emotion [9], contempt (Co), was subsequently added as one of the basic emotions [10]. Recognition of these emotions can be categorized into image-based [11,12,13,14,15,16,17,18,19,20,21,22,23,24,25,26,27,28,29,30,31,32,33,34,35,36,37] and video-based [38,39,40,41,42,43] approaches. Image-based approaches only use information about the static input image to determine the category of facial expression; on the other hand, except when the spatial features extracted from a static image are available, video-based approaches can also use temporal information of a dynamic image sequence to capture the temporal changes of facial appearance when some facial expression occurs. Considering that video-based approaches recognize facial expressions from large-scale image sequences, which inevitably lead to higher computational complexity, this work will focus on the image-based approach.

FER can also be divided into the traditional method [15,27,30,31,32,38,40], deep learning method [16,17,18,20,21,23,24,25,26,35,36,39,41,42,43], or a combination of the two [11,12,22,28,29,33,37]. Traditional FER systems usually involve facial representation and expression classification. Facial representation is crucial to the final accuracy of expression classification, which aims to make it more possible to distinguish the facial expressions. The majority of facial representations use handcrafted features, such as local binary patterns (LBP) [15], Gabor features [27], temporal patterns of oriented edge magnitudes (TPOEMs) [38], histogram of oriented gradients (HOG) [40], and bag-of-words (BoW) features [31] for FER. For expression classification, support vector machines (SVMs) are the most effective and common method, therefore, many studies [11,15,30,31,32,33,40] used SVM to build their classification algorithm. In the last few years, research on deep learning, especially on convolutional neural networks (CNNs), has made great progress in computer vision, including FER. Unlike traditional approaches, where features are defined manually and only shallow features can be obtained, deep learning methods stack a number of intermediate layers from input data to a classification layer and can automatically learn high-level features from a large amount of training data [44]. The high-level features are learned step-by-step for CNN, e.g., the first layer of CNN is usually responsible for extracting shallow features, which are then transformed into distinguishable mid-/high-level features through middle/rear convolutional layers. Therefore, the extraction of shallow features is very important for the CNN, as it can directly affect the accuracy of high-level features and the correctness of final classification. However, it is uncertain whether the first convolutional layer can provide complete and effective shallow features due to its huge number of uncertain parameters and its back-propagation mechanism [45]. Taking into account the excellent performance of traditional methods and uncertainty of the first convolutional layer of CNNs for shallow features extraction, we will consider using Gabor surface feature (GSF) [46], a facial representation method that combines the advantages of the LBP and Gabor algorithms instead of the first convolutional layer of CNNs to enhance the extraction of facial shallow features.

In addition, most literatures [12,22,23,33] have used standard convolutional layers, whose weights are shared across a whole face, to learn facial features. However, different regions of an aligned face have different local statistics, and the spatial stationarity assumption of convolution cannot hold [47]. To overcome this, the patch attention mechanism, where the weights are shared only within a local facial region, is employed to capture the local appearance changes of different facial regions.

**Figure 1:**
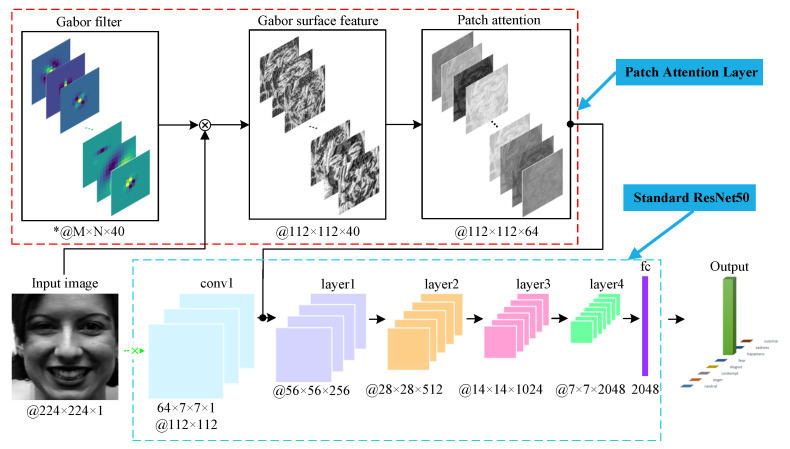
Framework of the proposed Patch Attention Layer (PAL) with the backbone network. “*” denotes that M and N are variable depending on the input Gabor parameters.

To sum up, we propose a Patch Attention Layer (PAL) of embedding handcrafted GSF, which can substitute the first convolutional layer of any standard CNN to capture certain shallow features. Then, we feed these outstanding and clearly representative shallow facial features to the remaining layers to achieve competitive results. Figure 1 illustrates the main idea of the proposed method. Firstly, we obtain GSF through the convolution of an input face image and Gabor filters; then, local features of GSF can be learned with the patch attention mechanism; finally, the output feature maps of PAL are fed into the remaining layers of standard CNNs for classification. In our experiment, we used ResNet50 [48] as the backbone CNN. Our major unique contributions are as follows:

- PAL, a simple plug-and-play module, is designed to learn relatively controllable and certain shallow facial features, then, its output features can be fed into any standard backbone CNN by skipping the first convolutional layer. This operation can significantly improve the performance of the whole network.
- According to the patch attention mechanism, we divide all GSFs into uniform patches. Unlike the traditional convolutional layer, where same weights are shared with whole feature maps, we make each patch has its own convolutional module to learn better distinguished local features for corresponding patches. In addition, we do not rely on landmark information used in [17,18,36,43,49], so we can reduce the risk caused by its accuracy.
- In terms of preprocessing, only face detection and alignment are done on the image, which is not as complicated as in some studies [16,50,51].
- We conduct experiments on four leading databases (CK+ [52], Oulu-CASIA [53], JAFFE [27], and RAF-DB [54]), which show that our approach has achieved competitive results compared with state-of-the-art approaches.

The remaining chapters are organized as follows. Section 2 reviews the most recent related work. Section 3 gives a description of the proposed PAL in detail. Section 4 presents the experimental settings, results, comparison with other approaches, and discussions. The conclusion is presented in Section 5.

## 2. Related Work

In this section, we mainly present previous works considering two issues that are related to ours, i.e., feature representation for FER and the patch attention mechanism.

### 2.1. Feature Representation for FER

FER performance highly depends on the quality of facial feature representation, which has attracted much attention from researchers. Facial expression features can be roughly divided into two categories: shallow features and high-level features. For the former, most of the existing works used different types of handcrafted features. Shan et al. [15] empirically evaluated facial representation based on a statistical local feature called LBP, experiments had shown that the LBP feature has a better, stable, and robust performance when the input facial images have different forms. To overcome the limitation that traditional LBP can lose the neighboring pixels related to different scales that can affect the texture of facial images, Yasmin et al. [30] proposed a new extended LBP method based on the bitwise “AND” operation of two rotational kernels to extract facial features. In view of satisfactory performance of the LBP operator, the CNNs that integrate advantages of the LBP have been developed [41,55,56]. Lyons et al. [27] used a multiscale, multiorientation set of Gabor filters to code facial expression images through comparing the similarity space derived from semantic ratings of the images by human observers with the one derived from Gabor representation; authors believed that the latter shows a significant degree of psychological plausibility. Cruz et al. [38] presented a novel descriptor TPOEM, which is an extension of the patterns of oriented edge magnitudes, by adding temporal information to represent facial images. Dahmane et al. [40] utilized dynamic dense grid-based HoG to extract facial features; the experiment showed that these features perform better than static uniform LBP implementation. Sikka et al. [31] applied a matured method, BoW—a technique highly successful in object and scene recognition—to FER, results showed that it is a successful method of knowledge transformation.

Researchers have used deep learning method to extract high-level features. Mollahosseini et al. [44] presented a new deep neural network (DNN) architecture to deal with the FER problem across seven well-known facial expression databases; the DNN has a good generalizability and accuracy. Wang et al. [35] proposed an oriented attention pseudo-siamese network that consists of a maintenance branch and an attention branch, this network not only grabs a global picture but can also concentrate on important local areas. Generally speaking, deep learning methods perform better than traditional ones, so deep learning methods have gradually become mainstream.

In order to obtain better performance, some researchers have tried a combination of the two features. Sun et al. [33] proposed a multichannel deep spatial–temporal feature fusion neural network whose inputs are gray-level emotional-face and optical flow features extracted from the changes between emotional-face and neural-face. References [11,12] employed a multimodal feature that consists of shallow features (facial key points, SIFT) and high-level features extracted by a CNN model, then SVM is applied to classification. Considering that handcrafted features and high-level features may have some similarities, references [22,28] proposed a general framework for embedding handcrafted feature constraints into a deep loss for feature learning. Hybrid methods, which can extract shallow invariant features of face images and high-level semantic features, have a great advantage for FER. Therefore, in this paper, a hybrid structure is used for recognition, i.e., PAL of embedding handcrafted GSF is responsible for extracting shallow features and standard CNN except first layer for high-level ones.

### 2.2. Patch Attention Mechanism

Humans have the ability to quickly filter out irrelevant information and lock in parts of interest when recognizing objects. Recently, this kind of attention mechanism has been successfully applied in FER [17,18,20,21,24,25,26,29,35,43,57]. Zhong et al. [21] divided a facial image into nonoverlapped patches to discover the common and specific patches that are important to discriminate all the expressions and only a particular expression, respectively; then, they discussed how different numbers of patches affect recognition performance. References [18,25] decomposed feature maps to sub-feature-maps to acquire local patches and then weighted them, subsequently using weighted patches to obtain the final feature representation, but these patches should be selected carefully. Instead of cropping small fixed patches, Wang et al. [17] presented a novel region attention network, which is fed to relatively large regions cropped in several ways to capture the importance of facial region. Zhao et al. [24] proposed a deep region and multilabel learning that is able to identify more specific regions for different Action Units (AUs) through a region layer that uses feed-forward functions to capture structural information in different facial regions. In a word, patch attention can increase the weight of the parts we are interested in, then, a better performance will be achieved just by focusing on the weighted parts.

## 3. Proposed Method

The proposed PAL contains two parts: GSF extraction model and patch attention model. Firstly, we use a set of Gabor filters to extract multiscale and multiorientation Gabor magnitude pictures (GMPs), which are then encoded to GSF. Secondly, GSF is divided into uniform patches, and each patch has its own convolutional component to capture local features. In this section, we will give a brief overview of the proposed PAL and then detail each part of it.

### 3.1. Overview

The sketch of our proposed PAL is illustrated in Figure 1. The input is an aligned gray face image, which is then convolved with 40 Gabor filters of size M × N × 1. It should be noted that M and N are variable depending on the input Gabor parameters, just like the calculation in Skimage toolbox (https://scikit-image.org/). In this paper, we use the notion 40 × M × N × 1 @ 112 × 112, where 112 × 112 denotes the output size of feature maps. After convolution, we can get 40 GMPs, which are then encoded to face representation GSF. Subsequently, we divide GSF into 49 nonoverlapped patches that will be fed into a patch attention to achieve local features. This part will be discussed in detail in Section 3.3. Finally, the output feature maps of PAL are fed into layer1 of ResNet50 to replace the first convolutional layer.

### 3.2. GSF Extraction Model

Considering the advantages of Gabor filters in face recognition [58], we use a set of Gabor filters [59] to extract multiscale and multiorientation face features. The definition of the Gabor filters is presented as

(1) $$\left\{\begin{array}{c} \psi_{\vec{k}}\left(\vec{r}\right)=\frac{{\vec{k}}^{2}}{\sigma^{2}}exp\left(-\frac{{\vec{k}}^{2}{\vec{r}}^{2}}{2\sigma^{2}}\right)\left[exp\left(i\vec{k}\vec{r}\right)\;-\;exp\left(-\frac{\sigma^{2}}{2}\right)\right]\\ \vec{k}={\left[k_{x}\;k_{y}\right]}^{T}={\left[k_{v}\cos\phi_{u}\;k_{v}\sin\phi_{u}\right]}^{T}\\ \vec{r}=[x^{\prime }\;y^{\prime }]=\;\left[x\cos\phi_{u}+y\sin\phi_{u}\;-x\sin\phi_{u}+y\cos\phi_{u}\right] \end{array}\right.,$$

where $k_{v}=2^{-\frac{v+2}{2}}\pi$ gives the frequency, $\phi_{u}=u\frac{\pi }{K}$ gives the orientation, and (x,y) represents a pixel in the image. Note that, in Equation (1), *u* and *v* control the orientation and scale of Gabor filters, respectively, and *K* represents the total number of orientation. In this paper, the parameters of Gabor filters are as follows: $\sigma =\frac{\pi }{2}$, v∈{0,1,2,3,4}, u∈{0,1,2,3,4,5,6,7}, K=8.

GSF, proposed in [46], which uses the 1st and 2nd derivatives information of GMPs, is employed for facial representation, since it is an effective texture analysis method in the spatial domain and takes advantage of both of Gabor and LBP. To extract GSF, firstly, GMPs (called G for short) should be calculated by convolving a face image with each of the 40 predefined Gabor filters described in Equation (1). Secondly, G are filtered by symmetric gradient operator along the two spatial dimensions *x* and *y*, then, the gradient pictures $G_{x},G_{y},G_{xx}$, and $G_{yy}$ can be obtained. Different from using [−1,0,1] as the gradient operator in paper [46], we use the Sobel operator [[−1,0,1],[−2,0,2],[−1,0,1]] instead because of its better performance on image denoising [60]. A set of Gs can be formulated as follows:

(2) $$\left\{\begin{array}{ccc} G & = & \cup_{1}^{40}\left(Input⊗Gabor_{i}\right)\\ G_{x} & = & G⊗sobel_{x}\\ G_{y} & = & G⊗sobel_{y}\\ G_{xx} & = & G_{x}⊗sobel_{x}\\ G_{yy} & = & G_{y}⊗sobel_{y} \end{array}\right.,$$

where ⊗ denotes the convolution operator and $sobel_{x}=sobel_{y}^{T}$. Finally, $G,G_{x},G_{y},G_{xx}$, and $G_{yy}$ are, respectively, binarized to the binary pictures $B,B_{x},B_{y},B_{xx}$, and $B_{yy}$. The GSF function $F_{gsf}$, which is encoded by the way similar to LBP, can be formulated as follows:

(3) $$F_{gsf}=2^{3}B+2^{2}B_{x}+2^{1}B_{y}+2^{0}B_{2},$$

where $B_{2}=B_{xx}+B_{yy}$. As an example, for each pixel z=(x,y) of G, its binary value is defined as

(4) $$B_{z}=\left\{\begin{array}{cc} 1, & \mathrm{if}\;G_{z}\geq Threshold_{g}\\ 0, & \mathrm{otherwise} \end{array}\right.,$$

where $Threshold_{g}$ is the median of pixel value of G. Therefore, $F_{gsf}$ is the feature map with the value ranging from 0 to 16, which is further transformed to interval [0,1] to make neural networks easier to converge.

### 3.3. Patch Attention Model

In a classic convolutional layer, the convolutional filters are shared by all regions of an entire input image and then generate feature maps. Under most conditions, this method is effective for dealing with feature extraction. However, for some tasks, e.g., FER, whose input is more structured and different regions follow different local statistics, the effectiveness of sharing the same set of filters for an entire image will decrease. Therefore, it would be better to process each local region with an independent filter because different local regions have various structures and texture information. Instead of employing a classic convolutional layer, the filters of region layer proposed in the paper [24] are shared only within the local facial region, and local appearance changes will be captured for different facial regions by adopting different filters for different regions. Nevertheless, the input of region layer is the feature maps generated by a convolution operation, which can only obtain uncertain shallow features. Thus, we use a traditional handcrafted texture face representation GSF instead. In addition, Global Average Pooling (GAP) [61] is employed in our method as it can effectively prevent overfitting and increase generalization ability.

**Figure 2:**
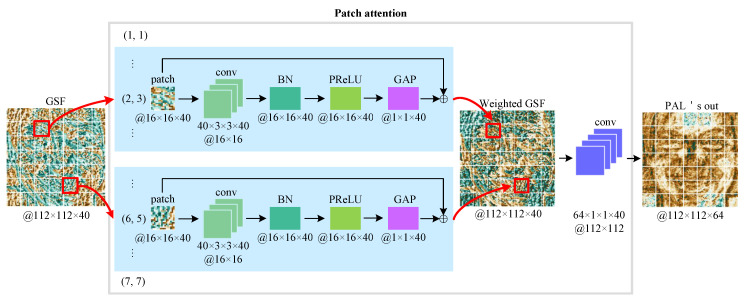
An illustration of the proposed patch attention model. GSF—Gabor Surface Feature; conv—Convolution operation; BN—Batch Normalization; PReLU—Parametric Rectified Linear Unit; GAP—Global Average Pooling.

Our patch attention model is illustrated in Figure 2. From the figure, we can know that the patch attention model has two parts: patch feature extraction and channel transformation. In the first part, we divide the GSF into uniform patches (a 7 × 7 grid), and then each patch is fed into a convolutional layer to learn its own feature maps individually. The feature maps are normalized using Batch Normalization (BN) [62] and passed through Parametric Rectified Linear Unit (PReLU) [63]. To obtain a weight to express the importance of each patch, GAP is then used. Finally, each patch is weighted by the computed weight with a residual structure to learn overcomplete features and avoid the vanishing gradient problem. In the second part, we use a 1×1 convolutional layer to match channels of layer1 of standard ResNet50 [48]; another benefit of this part is to increase the nonlinear ability of the network.

Mathematically speaking, let us suppose that $p_{i}$ denotes the input 16×16×40 *i*-th patch divided from 112×112×40 GSF; a corresponding weight $\alpha_{i}$, which represents the patch *i*’s importance, can be formulated as

(5) $$\alpha_{i}=f\left(p_{i}\right),$$

where *f* means the operations consist of a convolution, a BN, a PReLU, and a GAP operation.

Following the computation of attention weight, the *i*-th patch is then weighted, and outputs its weighted feature $\phi_{i}$ as follows:

(6) $$\phi_{i}=\alpha_{i}⊕p_{i},$$

where ⊕ is the element-wise addition.

Finally, a weighted GSF is reassembled from the weighted patches in the original order, which is then fed into a 1×1 convolutional layer to obtain PAL’s output referred to ${\mathrm{out}}_{pal}$, we can express ${\mathrm{out}}_{pal}$ like this:

(7) $${\mathrm{out}}_{pal}=Conv\left(Reassemble\left(\cup_{1}^{49}\phi \right)\right).$$

${\mathrm{out}}_{pal}$ is used as the final representation of the proposed PAL, we can feed it into any standard CNN except for the first convolutional layer.

## 4. Experiments

**Figure 3:**
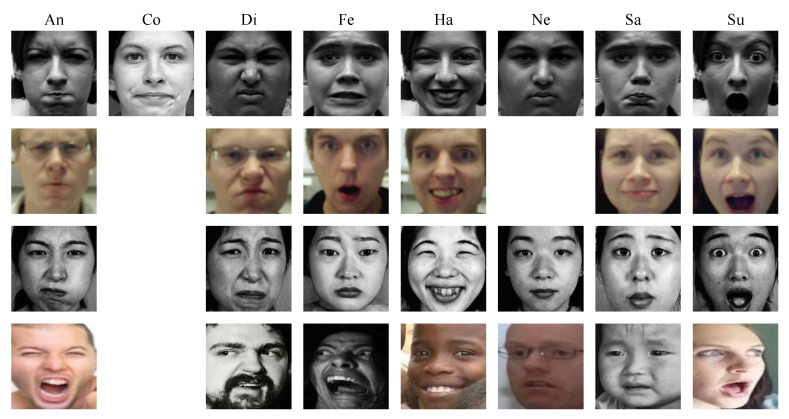
Samples of different expressions from four databases. From top to bottom is the Extended Cohn–Kanade database (CK+), Oulu-CASIA, Japanese Female Facial Expressions database (JAFFE), and RAF-DB. An, Co, Di, Fe, Ha, Ne, Sa, and Su stand for Anger, Contempt, Disgust, Fear, Happiness, Neutral, Sadness, and Surprise, respectively.

We evaluate the performance of our method on four well-known publicly available databases, namely, laboratory-controlled-condition databases such as CK+ [52], Oulu-CASIA [53], and JAFFE [27], and also an in-the-wild-condition database like RAF-DB [54]. The number of images per each expression used in our experiment is shown in Table 1, and Figure 3 demonstrates samples of different expressions from four databases. The details of the experiments and results will be expressed in the following sections.

### 4.1. Implementation Details

In our experiment, the HOG-based face detector in the Dlib toolbox (http://dlib.net/) and the Affine-Transformation-based face aligner in the Imutils package (https://github.com/jrosebr1/imutils) are used to detect and align faces, respectively. The aligned faces are then cropped and resized to the size of 224×224. We implement our method with a machine learning framework Pytorch 1.6 (https://pytorch.org/), and Pytorch Lightning 1.02 [64] is employed to ensure quick building and organization of our code. The experiments are carried out in the environment of Python 3.8 and operating system of Windows 10, where we use an Inter Xeon Gold 6134 3.2 GHz CPU and a NVIDIA QUADRO RTX8000 GPU with CUDA framework 10.2. For the backbone CNN, we use ResNet50 [48], which is initialized with the weights pretrained on the ImageNet database. To avoid overfitting, we apply a data augmentation during training that consists of random horizontal flipping with probability of 0.5 and color jitter with brightness of 0.4, contrast of 0.3, saturation of 0.25, and hue of 0.05. We use the Adam optimizer with a momentum of 0.9; a weight decay of 0.0005; a minibatch size of 32 for CK+, Oulu-CASIA, and RAF-DB, and 8 for JAFFE. The learning rate is initialized as 0.001 and decreased by a factor of 0.5 every 10 epochs. We stop training with 40 epochs in total. When training with Cross-Entropy loss, the flooding lever [65] is set at 0.03 to avoid zero training loss.

### 4.2. Comparison with the State-of-the-Art

The Extended Cohn–Kanade database (CK+) [52] is a extended vision based on the sCohn–Kanade database with 22% posed expression. The database includes 593 image sequences recorded from 123 subjects ranging from 18 to 30 years old. For each sequence, the intensity of expression starts from neutral to the peak. Among these sequences, only 327 sequences from 118 subjects have seven basic facial expressions and only the last frame of each sequence is labeled. We select the last three frames and the first frame of each sequence to compose our experimental database. We further split the sequences into 10 subject-independent subsets for 10-fold cross-validation by sampling in ID ascending order, which is the same as the previous works [19,22,28,33]. In each time, we use nine subsets for training and the remaining one is used for validation.

In Table 2, we compare our method with current state-of-the-art works, which used traditional, deep learning, or a combination of the both methods. The average accuracy of 10 runs for seven-class and eight-class are reported. Among the many previous works, some works such as STRNN [42], LBVCNN [41], TPOEM [38], PHRNN-MSCNN [39], and SAANet [43] used image sequence as their experimental data, while others used a static image. Although Specific preprocessing [16], ALAW [22], Feature loss [28], OAENet [35], and S-DSRN [23] used seven expressions, contempt expression is replaced with neural. Note that, in order to make a fair comparison, seven basic expressions with and without neutral are used for our experiment. The studies [43] and [33] achieved the best performance for seven-class and eight-class, respectively. However, [43] employed image sequence as input, which means a lot of computation. Extra temporal feature extraction in [33] needs a much more complex and wider network structure. For both cases, our method significantly outperforms all others, achieving 99.69% vs. the previous best of 99.54% for seven-class, and 98.93% vs. 98.38% for eight-class. These are now the new state-of-the-art performances as far as we know.

**Figure 4:**
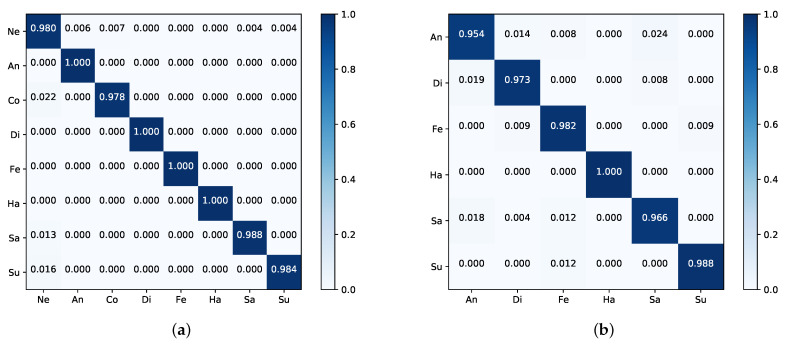
The confusion matrices of our PAL on the (**a**) CK+ for the eight-class and (**b**) Oulu-CASIA. The darker the color, the higher the accuracy.

Figure 4a is the confusion matrix on the CK+; here, we only express the eight-class FER problem. From Figure 4a, we can know that our method performs well on anger, disgust, fear, and happiness; contempt expression is the most difficult to classify due to having the lowest recognition rate. One possible reason is that contempt expression has the least amount in CK+, in addition, the way people express it is very elusive.

The Oulu-CASIA database [53] contains data captured under three different illumination conditions (dark, strong, and weak) using two types of cameras (near infrared and visible light). It consists of six basic facial expressions (apart from contempt) from 2880 image sequences of 80 subjects between 23 to 58 years old. Similar to the CK+, all sequences begin with neutral expression and end with the peak one. We only apply the 480 sequences with strong condition captured by a visible light camera to our experiment. The last three frames of each sequence are selected for our experiment. Similar to the experimental setting in CK+, a 10-fold subject-independent cross-validation method is performed.

Table 3 reports the comparison between our method with state-of-the-art algorithms on Oulu-CASIA. Our PAL method achieves the best performance and outperforms the previous best video-based work SAANet [43] by 9.24%. For the image-based method, Attention-based CNN [37], our model outperforms it by 2.94%. The confusion matrix in Figure 4b expresses that happiness expression is very easy to be recognized, while anger and sadness show relatively low performance.

The RAF-DB [54] is a large-scale in-the-wild expression database collected from the internet. It is annotated with basic or compound expressions by 40 independent trained human coders. In our experiment, only images with six basic facial expressions (apart from contempt) as well as neutral are used. We employ the specified training and testing sets provided by the database, including 12,271 training and 3068 test images.

**Figure 5:**
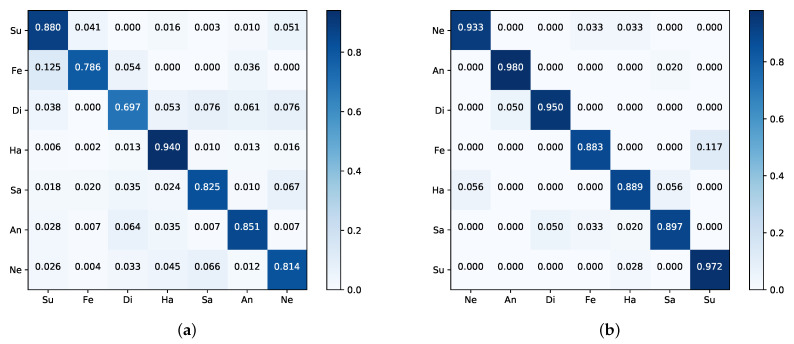
The confusion matrices of our PAL on the (**a**) RAF-DB and (**b**) JAFFE. The darker the color, the higher the accuracy.

The performance comparison with RAF-DB is shown in Table 4, one can see that the proposed method has slight deterioration compared with the SCN [66], which outperforms other algorithms in terms of accuracy by suppressing the uncertainties of facial expression data. However, compared to the backbone ResNet50 in the same setting, our method surpasses it by 4.43% because PAL can enhance the model’s ability of focusing on local patches of interest. Figure 5a shows the confusion matrix of our method, it indicates that happiness has the highest accuracy and disgust is the most difficult to classify.

The Japanese Female Facial Expressions database (JAFFE) [27] consists of 213 images from 10 Japanese female subjects. In this database, each image is labeled as one of six basic (except contempt) and neutral facial expressions. The size of each image is 256×256 with 8-bit precision for gray-scale values. Similar to the experimental setting in the CK+, a 10-fold subject-independent cross-validation method is performed.

As shown in Table 5, our method achieves better performance and shows high results on JAFFE for seven-class. The Attention-based CNN method [37], which features the highest accuracy of the methods shown in Table 5, is not as good as ours for the CK+ and Oulu-CASIA. Note that the work [32] achieved an accuracy of 94.8% for six-class by a new face descriptor, namely, local directional ternary pattern; however, for seven-class, we achieved an 0.18% improvement compared to theirs. The confusion matrix is reported in Figure 5b, which indicates that our method performs well in anger, disgust, and surprise, while fear expression has the lowest recognition rate, which is mostly confused with surprise.

### 4.3. Cross-Database Evaluation

The best way to evaluate generalization ability is the cross-database experiment. To perform such an experiment, we train the model on the CK+ and test it on the JAFFE. The result in a cross-database experiment is computed as an average of the ten runs. In this experiment, no images from the JAFFE are used during the training. The recognition results compared with other methods for cross-database experiment are shown in Table 6.

Although our proposed method achieves a competitive recognition accuracy of 46.48%, one can see that generalization performance is much lower than the results obtained within the CK+ experiment. The same situations were encountered in previous papers [12,16,29,38]. The low accuracy reported in Table 6 can be explained in terms of difference between knowledge, i.e., there is a big gap in the learnable features of images for the two databases.

## 5. Conclusions

In this paper, we put forward a novel method based on patches of interest for automatic FER. We designed the Patch Attention Layer (PAL) with embedded handcrafted GSF to learn certain local shallow facial features of each patch on face images. Considering its excellent performances for face representation, a multiscale and multiorientation GSF is first obtained with a set of Gabor filters for extracting shallow features. Motivated by humans’ ability to quickly filter out irrelevant information and lock in parts of interest, patch attention mechanism, whose weights are shared only within a local facial patch, is employed to capture local appearance changes of different facial patches for GSF. The strategy that each patch has its own convolutional module to learn distinguishable local features for corresponding patches can increase the weight of parts we are interested in and achieve better performance just by focusing on weighted parts. Lastly, the weighted shallow features are fed into the remaining convolutional layers to capture high-level features. Our method can be carried out directly on a static image without relying on facial landmarks, and only a simple preprocessing method with face detection and the alignment is executed. We evaluate our method on ideal-condition databases such as the CK+, Oulu-CASIA, and JAFFE, and an in-the-wild-condition database, RAF-DB, experimental results show that our method is competitive or even better compared to the state-of-the-art approaches. Although competitive results can be obtained with the proposed model, there is still much room for improvement. In the future, we plan to investigate more generalized pattern recognition methods for FER in the wild and consider real-time requirements in practical applications.

## Figures and Tables

**Table 1:** Number of images per each expression in the Extended Cohn–Kanade database (CK+), Oulu-CASIA, Japanese Female Facial Expressions database (JAFFE), and RAF-DB. An, Co, Di, Fe, Ha, Ne, Sa, and Su stand for Anger, Contempt, Disgust, Fear, Happiness, Neutral, Sadness, and Surprise, respectively.

Databases	An	Co	Di	Fe	Ha	Ne	Sa	Su	Total
CK+	135	54	177	75	207	327	84	249	1308
JAFFE	30	-	29	32	31	30	31	30	213
Oulu-CASIA	240	-	240	240	240	-	240	240	1440
RAF-DB	867	-	877	355	5957	3204	2460	1619	15,339

**Table 2:** Performance comparison with different methods on the CK+. Symbol “-” denotes not reported. Symbol “*” denotes that contempt expression is replaced with neural. “10F” denotes “10-fold cross-validation”. “T” and “DL” denote “Traditional-based” and “Deep-learning-based”, respectively.

Method	Data	Classes	Protocol	Category	Recognition Rate (%)
LDTP 2017 [32]	Three peak	7	-	T	94.2
SIFT-CNN 2019 [12]	The peak	7	8F	T + DL	94.13
STRNN 2018 [42]	Video-based	7	10F	DL	95.4
LBVCNN 2019 [41]	Video-based	7	10F	DL	97.38
TPOEM 2018 [38]	Video-based	7	10F	T	92.91
PHRNN-MSCNN 2017 [39]	Video-based	7	10F	DL	98.5
SAANet 2020 [43]	Video-based	7	10F	DL	99.54
Specific preprocessing 2017 [16]	Three peak	7 *	8F	DL	95.79
ALAW 2019 [22]	Three peak	7 *	10F	T + DL	97.35
Feature loss 2018 [28]	Three peak	7 *	10F	T + DL	97.35
OAENet 2020 [35]	The peak	7 *	10F	DL	98.5
S-DSRN 2018 [23]	Five peak	7 *	15F	DL	99.23
MSFLBP 2020 [30]	Image-based	7	10F	T	99.12
Multimodal feature 2020 [11]	The peak	7	-	T + DL	94.41
Attention-based CNN 2020 [37]	Three peak	7	5F	T + DL	98.68
DeRL 2018 [34]	Three peak	7	10F	DL	97.3
**Ours (RAL)**	Three peak	7	10F	T + DL	**99.69**
BDBN 2014 [14]	Three peak	8	8F	DL	96.7
FN2EN 2017 [19]	Three peak	8	10F	DL	96.8
MDSTFN 2019 [33]	Three images	8	10F	T + DL	98.38
MFP-CNN 2020 [36]	Image-based	8	10F	DL	98.07
**Ours (RAL)**	Three peak	8	10F	T + DL	**98.93**

**Table 3:** Performance comparison with different methods on the Oulu-CASIA. Symbol “-” denotes not reported.

Method	Data	Protocol	Category	Recognition Rate (%)
LBVCNN 2019 [41]	Video-based (-)	10F	DL	82.41
PHRNN-MSCNN 2017 [39]	Video-based (Strong -)	10F	DL	86.25
SAANet 2020 [43]	Video-based (-)	10F	DL	88.33
ALAW 2019 [22]	Three peak (Strong VIS)	10F	T + DL	85.83
FN2EN 2017 [19]	Three peak (Strong VIS)	10F	DL	87.71
DeRL 2018 [34]	Three peak (Strong VIS)	10F	DL	88
Attention-based CNN 2020 [37]	Three peak (-)	5F	T + DL	94.63
**Ours (RAL)**	Three peak (Strong VIS)	10F	T + DL	**97.57**

**Table 4:** Performance comparison with different methods on the RAF-DB.

Method	Classes	Category	Recognition Rate (%)
gACNN 2018 [18]	7	DL	85.07
DLP-CNN 2019 [67]	7	DL	84.13
Soft-label CNN 2019 [68]	7	DL	86.31
SCN 2020 [66]	7	DL	**87.03**
RAN 2020 [17]	7	DL	86.9
OAENet 2020 [35]	7	DL	86.5
Backbone (ResNet50)	7	DL	82.37
**Ours (PAL)**	7	T + DL	86.8

**Table 5:** Performance comparison with different methods on the JAFFE. Symbol “-” denotes not reported. “LOSO” denotes “Leave One Subject Out”.

Method	Classes	Protocol	Category	Recognition Rate (%)
LDTP 2017 [32]	6	-	T	94.8
	7			93.2
Specific preprocessing 2017 [16]	6	LOSO	DL	56.44
	7			53.57
Feature loss 2018 [28]	7	10F	T + DL	83.57
Attention-based CNN 2020 [37]	7	5F	T + DL	**98.52**
Multimodal feature 2020 [11]	7	LOSO	T + DL	91.8
**Ours (PAL)**	7	10F	T + DL	93.38

**Table 6:** Cross-database evaluation on the JAFFE with models trained on the CK+.

Method	Classes	Recognition Rate (%)
SVM based on LBP 2009 [38]	7	41.3
Specific preprocessing 2017 [16]	7	37.36
SIFT-CNN 2019 [12]	7	**48.90**
Feature optimization model 2017 [29]	7	46.01
**Ours (PAL)**	7	46.48

## Data Availability

Data sharing not applicable.
